# A comprehensive evaluation of pathogenic mutations in primary cutaneous melanomas, including the identification of novel loss-of-function variants

**DOI:** 10.1038/s41598-019-53636-x

**Published:** 2019-11-19

**Authors:** Ivana Ticha, Jan Hojny, Romana Michalkova, Ondrej Kodet, Eva Krkavcova, Nikola Hajkova, Kristyna Nemejcova, Michaela Bartu, Radek Jaksa, Miroslav Dura, Madiha Kanwal, Andra S. Martinikova, Libor Macurek, Petra Zemankova, Zdenek Kleibl, Pavel Dundr

**Affiliations:** 1 Institute of Pathology, First Faculty of Medicine, Charles University and General University Hospital in Prague, Prague, Czech Republic; 2 Department of Dermatology and Venereology, First Faculty of Medicine, Charles University and General Hospital in Prague, Prague, Czech Republic; 3 Institute of Anatomy, First Faculty of Medicine, Charles University, Prague, Czech Republic; 4 BIOCEV, Charles University, First Faculty of Medicine, Vestec, Czech Republic; 5 Cancer Cell Biology, Institute of Molecular Genetics of the Czech Academy of Sciences, Prague, Czech Republic; 6 Institute of Biochemistry and Experimental Oncology, First Faculty of Medicine, Charles University, Prague, Czech Republic

**Keywords:** Biomarkers, Melanoma, Predictive markers, Melanoma

## Abstract

The most common histological subtypes of cutaneous melanoma include superficial spreading and nodular melanoma. However, the spectrum of somatic mutations developed in those lesions and all potential druggable targets have not yet been fully elucidated. We present the results of a sequence capture NGS analysis of 114 primary nodular and superficial spreading melanomas identifying driver mutations using biostatistical, immunohistochemical and/or functional approach. The spectrum and frequency of pathogenic or likely pathogenic variants were identified across 54 evaluated genes, including 59 novel mutations, and the newly identified *TP53* loss-of-function mutations p.(L194P) and p.(R280K). Frequently mutated genes most commonly affected the MAPK pathway, followed by chromatin remodeling, and cell cycle regulation. Frequent aberrations were also detected in the genes coding for proteins involved in DNA repair and the regulation and modification of cellular tight junctions. Furthermore, relatively frequent mutations were described in *KDR* and *MET*, which represent potential clinically important targets. Those results suggest that with the development of new therapeutic possibilities, not only *BRAF* testing, but complex molecular testing of cutaneous melanoma may become an integral part of the decision process concerning the treatment of patients with melanoma.

## Introduction

Cutaneous melanoma (CM) is a malignant tumor with an increasing incidence worldwide. The most common cutaneous melanoma subtypes include superficial spreading melanoma (SSM) and nodular melanoma (NM)^1^.

Germline mutations in a number of genes have already been associated with an increased risk of familial CM (including *BAP1*, *CDKN2A*, *CDK4*, *MITF*, *TERT*, *POT1*). Several studies have also analyzed a spectrum of somatic mutations in distinct melanoma subtypes and identified the key CM drivers that comprised mutations in *BRAF*, *RAS* (mainly *NRAS*, but mutations in *HRAS* and *KRAS* are responsible for <1%), *NF1*, *TP53*, *CDKN2A*, *PTEN*, *IDH1*, *ARID2*, *RAC1*, *RB1*, and *PPP6C*^2–7^. Systematical analyses of genomic data retrieved from publicly available datasets, especially from the largest available dataset at the TCGA Research Network (https://www.cancer.gov/tcga), identified other genes associated either with melanoma susceptibility, development or progression^8–11^. However, a majority of the previously published studies dealing with the identification of frequently-mutated melanoma genes lacked a detailed description of how the evaluation of pathogenicity of the detected mutations was performed (especially when it comes to novel, non-curated variants, and single nucleotide variants with a conflicting interpretation). Therefore, data on the prevalence of pathogenic and likely pathogenic (class 4 and 5) mutations and their contribution to melanomagenesis has not yet been analyzed systematically.

In this study, targeted next generation sequencing (NGS) analysis was performed using a custom-designed panel (spanning 219 kbp) in a sample set of 114 primary CMs from Czech patients, all with complete clinico-pathological data. The goal of the study was (i) to perform NGS analysis and a comprehensive biostatistical evaluation of the pathogenicity of the detected variants, (ii) to describe the spectrum and frequency of somatic class 4/5 mutations in primary CMs, (iii) to validate the biostatistical algorithm for pathogenicity prediction, and (iv) to analyze the relationship between frequently mutated genes and clinico-pathological variables or disease outcome, including disease-free survival (DFS), local recurrence-free survival (LFS), and metastasis-free survival (MFS). The results should help in further uncovering of the spectrum and frequency of the clinically important variants in CM.

## Results

### Variants detected in primary CMs

**Figure 1:**
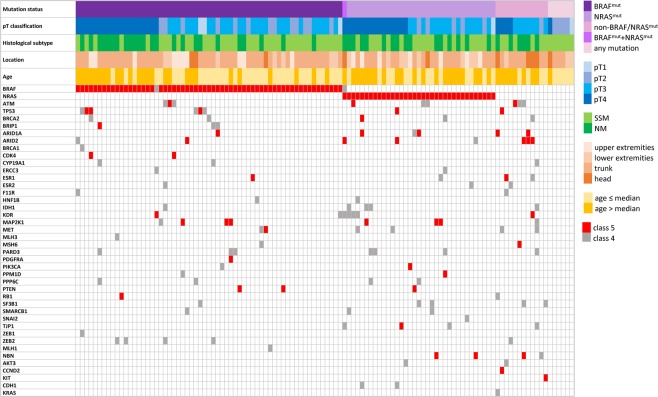
Spectrum of genes with detected pathogenic or possible pathogenic variants in 114 primary cutaneous melanomas with respect to their clinico-pathological characteristics. Cells in red indicate pathogenic mutation, cells in gray indicate likely pathogenic mutation.

NGS analyses of primary CMs from 114 patients (Table 1) revealed on average 288 variants detected per sample, including all the detected synonymous and non-synonymous variants with variant allele frequency (VAF) >10%. Implementation of high-stringent probe design (chosen to eliminate non-specific probe binding) led to the exclusion of 18 genes from further evaluation due to low coverage in a majority of samples (Supplementary Data 1). The prioritized variants in 54 evaluated genes were subsequently processed (for detailed information see Methods and Supplementary Table 1). The biostatistical algorithm filtered out 142 different benign or likely benign variants in 41 genes (33 VUS and 109 novel missense variants, mainly novel variants detected in *ATM*, *BRCA2*, *ESR1*, *KDR*, *PPM1D*, *SF3B1*, and *TJP1*). Altogether, we found 144 different class 4/5 mutations in 42 out of 54 evaluated genes (Table 2). The spectrum of mutations included 59 novel mutations in 31 genes (underlined in Table 2). Among these were 16 protein truncation mutations in *ARID1A*, *ARID2*, *ATM*, *CCND2*, *ESR1*, *KDR*, *MET*, *PDGFRA*, *PTEN*, and *TJP1*, 8 splice mutations in the consensus splice site in *AKT3*, *ARID2*, *HNF1B*, *PPM1D*, *RB1*, and *TP53*, one in-frame mutation (*ZEB2*), one frameshift mutation leading to a loss of the termination codon (*PARD3*) and 33 missense class 4/5 mutations in 23 genes. The most prevalent pathogenic missense *BRAF* and *NRAS* mutations were mutually exclusive, except for one case with *NRAS* p.(G13C) and *BRAF* p.(G466E) variants, which are both outside the hot-spot codons and of uncertain clinical impact. The *BRAF* mutations (*BRAF*^*mut*^) were present in 62/114 (54.4%) cases, of those 57 (91.9%) harbored a mutation in the V600 codon. The *NRAS* mutations (*NRAS*^*mut*^) were detected in 35/114 (30.7%) cases, and the alteration of the Q61 codon was present in 34 (97.1%) cases. Other genes frequently affected by class 4/5 mutations were *ARID1A*, *ARID2*, *ATM*, *KDR*, *MAP2K1*, *MET*, *PARD3*, and *TP53* (>5% of cases), and *BRCA2*, *ESR1, IDH1*, *NBN*, *PPP6C, PTEN*, *SF3B1, TJP1* and *ZEB2* (3–5% of cases). At least one class 4/5 variant was identified in 108/114 (95%) of the samples. In 12 non-*BRAF*^*mut*^*/*non-*NRAS*^*mut*^ samples, class 4/5 mutations affected genes *ARID1A*, *ARID2*, *AKT3*, *ATM*, *BRCA2, CCND2*, *CYP19A1*, *ESR1*, *ESR2, F11R, IDH1, KDR*, *KIT*, *MAP2K1*, *MET*, *MSH6, NBN, PARD3*, *SF3B1*, *TJP1*, *TP53*, and *ZEB2*. In 6/114 (5%) patients no class 4/5 mutation was found (Fig. 1). The number of mutations/mutated genes per sample ranged between 0 to 6 (mean = 2.4/2.3, median = 2). We identified multiple mutations (>1) in 69/114 (60.5%) samples (Fig. 1). In samples with mutually-exclusive *BRAF* and *NRAS* mutations, we identified an additional mutation in 34/62 (54.8%) *BRAF*^*mut*^ and in 26/35 (74.3%) *NRAS*^*mut*^ cases, respectively. Interestingly, one melanoma which developed a novel *NRAS* non-hot-spot mutation p.(P140S) predicted by *in silico* approach to be benign also carried a pathogenic hot-spot *KRAS* mutation p.(G12C). Furthermore, the *in silico* analyses algorithm suggested the pathogenicity of 15 missense variants in 10 genes (*ATM*, *BRCA2*, *BRIP1*, *CDH1*, *IDH1*, *MET*, *NBN*, *RB1*, *SF3B1*, and *TP53*) which are catalogued in the ClinVar with conflicting interpretation or uncertain significance. The pathogenicity of *ARID1A* and *TP53* mutations was also confirmed by immunohistochemical and functional assays. We did not find any class 4/5 mutation in 12 of 54 evaluated genes, namely in *BARD1*, *JAM2*, *JAM3*, *MAP2K1*, *MDM2*, *MITF*, *MSH2*, *MYC*, *PALB2*, *POT1*, *RAD51C*, and *RAD51D*.

**Table 1:** Clinico-pathological characteristics of the 114 patients with primary cutaneous melanoma.

Characteristic	Group	N (%)
**Sex**	Male	69 (60.5%)
	Female	45 (39.5%)
**Age range: 24–93**	≤66	58 (50.9%)
(mean/median = 62/66 years)	>66	56 (49.1%)
**Classification (tumor stage)**	pT1 (≤1 mm)	5 (4.4%)
	pT2 (>1–2 mm)	23 (20.2%)
	pT3 (>2–4 mm)	48 (42.1%)
	pT4 (>4 mm)	38 (33.3%)
**Location**	Head	10 (8.8%)
	Trunk	69 (60.5%)
	Upper extremities	17 (14.9%)
	Lower extremities	18 (15.8%)
**Histological subtype**	SSM	68 (59.6%)
	NM	46 (40.4%)
**Ulceration**	No	60 (52.6%)
	Yes	54 (47.4%)
**Regression**	No	93 (81.6%)
	Yes	21 (18.4%)
**Sentinel nodes**	No	43 (37.7%)
	Yes	71 (62.3%) - 19 positive (26.7%)
**TILs**^†^	Absent	7 (9.5%)
(scoring system by Clark)	Non-brisk	50 (67.5%)
	Brisk	17 (23.0%)
**Death due to melanoma**^‡^	No	71 (67.6%)
^§^mean/median DFS = 34/24 months	Yes	34 (32.4%)
**Local recurrence**^‡^	No	88 (83.8%)
^§^mean/median LFS = 22/10 months	Yes	17 (16.2%)
**Metastasis**^‡^	No	81 (77.1%)
^§^mean/median MFS = 25/18 months	Yes	24 (22.9%)

^†^Information is missing for several cases, ^‡^data is based on 105 cases without targeted therapy. ^§^Mean and median of months was calculated only for cases with respective events. DFS – disease-free survival, LFS – local recurrence-free survival, MFS – metastasis-free survival, NM – nodular melanoma, SSM - superficial spreading melanoma, TIL – tumor infiltrating lymphocytes. TILs were assessed using the scoring system by Clark as described elsewhere^46^.

**Table 2:** The summary of the pathogenic and likely pathogenic variants detected in the 114 patients with primary cutaneous melanoma.

Gene^†^	Variant	Case number^‡^ (freq.)	Gene^†^	Variant	Case number^‡^ (freq.)	Gene^†^	Variant	Case number^‡^ (freq.)
**BRAF**	G466E	1 (0.9%)	**CCND2**	p.W139X	1 (0.9%)	**NBN**	p.W2X	1 (0.9%)
(total 62)	G469E	1 (0.9%)	(total 1)			(total 4)	p.E309K	1 (0.9%)
	L597S	1 (0.9%)	**CDH1**	p.F767S	1 (0.9%)		p.E736X	1 (0.9%)
	V600E	46 (40%)	(total 2)	p.G877R	1 (0.9%)		p.D742H	1 (0.9%)
	V600K	10 (8.7%)	**CDK4**	p.R24C	1 (0.9%)		p.K219delinsNLfs	1 (0.9%)
	V600R	1 (0.9%)	(total 2)	p.R24H	1 (0.9%)	**PARD3**	p.P170S	1 (0.9%)
	K601E	2 (1.8%)	**CYP19A1**	p.R159C	1 (0.9%)	(total 7)	p.S292T	3 (2.6%)
**NRAS**	G13C	1 (0.9%)	(total 3)	p.E335K	1 (0.9%)		p.R546C	1 (0.9%)
(total 35)	Q61R	16 (14%)		p.L451F	1 (0.9%)		p.G1326R	1 (0.9%)
	Q61K	15 (13.2%)	**ERCC3**	p.P149S	1 (0.9%)		p.X1357SfsX4	1 (0.9%)
	Q61L	2 (1.8%)	(total 2)	p.G402C	1 (0.9%)	**PDGFRA**	p.W586X	1 (0.9%)
	Q61H	1 (0.9%)	**ESR1**	p.R157X	1 (0.9%)	(total 1)		
**AKT3**	p.Y269S	1 (0.9%)	(total 4)	p.G415E	1 (0.9%)	**PIK3CA**	p.A615V	1 (0.9%)
(total 2)	p.R270C	1 (0.9%)		p.E397Sfs	1 (0.9%)	(total 2)	p.H1048R	1 (0.9%)
	c.1355–1G>A, spl.	1 (0.9%)		p.H488Y	1 (0.9%)	**PPM1D**	p.Q524X	1 (0.9%)
**ARID1A**	p.Q583X	1 (0.9%)	**ERS2**	p.D194N	1 (0.9%)	(total 2)	c.-232–2G>T, spl.	1 (0.9%)
(total 6)	p.Q1188X	1 (0.9%)	(total 3)	p.R221G	1 (0.9%)	**PPP6C**	p.H151Y	1 (0.9%)
	p.K1230Mfs	1 (0.9%)		p.T299I	1 (0.9%)	(total 4)	p.R301C	3 (2.6%)
	p.P1618S	1 (0.9%)	**F11R**	p.G105E	1 (0.9%)	**PTEN**	p.K6X	1 (0.9%)
	p.R1721X	1 (0.9%)	(total 2)	p.R286Q	1 (0.9%)	(total 3)	p.G209X	1 (0.9%)
	p.Q1894X	1 (0.9%)	**HNF1B**	p.S242F	1 (0.9%)		p.Y29delinsX	1 (0.9%)
	p.V2244G	1 (0.9%)	(total 2)	c.1340–1G>A, spl.	1 (0.9%)		c.1026 + 2T>G, spl.	1 (0.9%)
**ARID2**	p.R314C	1 (0.9%)	**IDH1**	p.R82K	1 (0.9%)	**RB1**	p.R251Q	1 (0.9%)
(total 8)	p.Q397X	1 (0.9%)	(total 5)	p.R132C	2 (1.8%)	(total 2)	c.2106+1G>A, spl.	1 (0.9%)
	p.Q490X	1 (0.9%)		p.Y183C	2 (1.8%)	**SF3B1**	p.P409S	1 (0.9%)
	p.E533X	1 (0.9%)	**KDR**	p.W485X	1 (0.9%)	(total 5)	p.R568C	1 (0.9%)
	p.R542X	1 (0.9%)	(total 7)	p.W610X	1 (0.9%)		p.R625H	1 (0.9%)
	p.Q782X	1 (0.9%)		p.R1032Q	2 (1.8%)		p.K666M	1 (0.9%)
	p.Q1313X	1 (0.9%)		p.W1096X	1 (0.9%)		p.E902K	1 (0.9%)
	p.E258Mfs34X	1 (0.9%)		p.L1156S	1 (0.9%)		p.H1210Y	1 (0.9%)
	c.5061 + 2T>A, spl.	1 (0.9%)		p.D1241N	1 (0.9%)	**SMARCB1**	p.P48L	1 (0.9%)
	c.1023 + 5G>A, spl.	1 (0.9%)	**KIT**	p.W557R	1 (0.9%)	(total 3)	p.A144V	1 (0.9%)
	c.187–1G>A, spl.	1 (0.9%)	(total 1)				p.S274F	1 (0.9%)
**ATM**	p.Q1003X	1 (0.9%)	**KRAS**	p.G12C	1 (0.9%)	**SNAI2**	p.L256V	1 (0.9%)
(total 7)	p.R1730X	1 (0.9%)	(total 1)			(total 1)		
	p.S1905delinsITfs	1 (0.9%)	**MAP2K1**	p.C121G	1 (0.9%)	**TJP1**	p.P1257S	1 (0.9%)
	p.E2014K	1 (0.9%)	(total 8)	p.P124S	5 (4.3%)	(total 4)	p.R1356X	1 (0.9%)
	p.G2023R	2 (1.8%)		p.P124R	1 (0.9%)		p.S1468F	2 (1.8%)
	p.L2447S	1 (0.9%)		p.S228F	1 (0.9%)	**TP53**	p.L194P	1 (0.9%)
	p.T2743M	1 (0.9%)	**MET**	p.Y126X	1 (0.9%)	(total 8)	p.R196X	1 (0.9%)
**BRCA1**	p.Q94X	1 (0.9%)	(total 6)	p.P657S	1 (0.9%)		p.R273P	1 (0.9%)
(total 1)				p.P664A	2 (1.8%)		p.G245D	1 (0.9%)
**BRCA2**	p.H2417Qfs	1 (0.9%)		p.G896E	1 (0.9%)		p.R280K	1 (0.9%)
(total 5)	p.P2532L	1 (0.9%)		p.T1010I	1 (0.9%)		c.75–1G>T, spl.	1 (0.9%)
	p.S2670L	1 (0.9%)	**MLH1**	p.P309A	1 (0.9%)		c.782+1G>C, spl.	1 (0.9%)
	p.A2730V	1 (0.9%)	(total 1)				c.919+1G>A, spl.	1 (0.9%)
	p.S2807L	1 (0.9%)	**MLH3**	p.C320G	1 (0.9%)	**ZEB1**	p.D1024N	1 (0.9%)
**BRIP1**	p.R162X	1 (0.9%)	(total 1)			(total 1)		
(total 3)	p.S624L	1 (0.9%)	**MSH6**	p.H437Y	1 (0.9%)	**ZEB2**	p.P425L	1 (0.9%)
	p.R814C	1 (0.9%)	(total 2)	c.3647–1G>A, spl.	1 (0.9%)	(total 5)	p.V463A	1 (0.9%)
							p.P506del	1 (0.9%)
							p.V533A	1 (0.9%)
							p.G1068D	1 (0.9%)

^†^Total count of patients with at least one mutation in the respective gene, ^‡^frequencies of the variant are related to the total number of patients; spl. – splice, fs – frameshift; novel variants are underlined.

### Germline mutations

We identified germline mutation in three melanoma patients. The first one was a germline *ATM* c.8228C > T, p.(T2743M) variant (found alongside the somatic *BRAF* p.(V600E), and *CDK4* p.(R24H) mutations) in a male patient diagnosed at the age of 32 years with pT3 primary NM located at forearm. The second one was a germline *MLH3* mutation c.958T > G, p.(C320G) (found together with somatic pathogenic mutation in *BRAF* p.(G469E), and a likely pathogenic mutation in *ZEB2* p.(V463A)) which was identified in a female patient diagnosed with primary pT4 NM with ulceration on the back at the age of 84. Finally, there was a germline, likely pathogenic *IDH1* mutation c.245G > A, p.(R82K) (found alongside somatic, likely pathogenic mutations in *BRAF* p.(V600E), *ATM* p.(E2014K), *ESR2* p.(T299I), and *ZEB2* p.(G1068D)) detected in one female patient diagnosed with pT2 SSM on a lower extremity at the age of 76.

### Primary melanoma pathways

The majority of the affected genes codes for proteins which are involved in RAS signaling (*BRAF*, *NRAS*, *ESR1, ESR2*, *MAP2K1*, *MET*, *KDR*, and *PTEN*), DNA damage response and cell cycle regulation (*ATM*, *BRCA1*, *BRCA2*, *BRIP1*, *KDR*, *NBN*, *PPM1D*, *PPP6C*, *PTEN*, *SMARCB1*, and *TP53*), chromatin remodeling (*ARID1A*, *ARID2*, *SMARCB1*), or tight junction regulation (*PARD3*, *TJP1*, *ZEB2*). Another frequently affected genes not assigned into main functional pathways were *CYP19A1* (cytochrome P450 family monooxygenase), *ESR1* (nuclear hormone receptor signaling pathway), *IDH1* (enzyme in citrate cycle), or *SF3B1* (splicing factor 3B subunit, RNA splicing).

### Validation of the *in silico* prediction

Only the genes affected by mutations with an already known impact and those where an optimized functional and/or IHC analysis was available were chosen for this validation. We performed immunohistochemical (IHC) analysis of ARID1A and p53 protein expression in tissue sections from samples with mutations. Functional *in vitro* assessment of the detected *TP53* variants was also performed in order to validate the utility of the *in silico* prediction of mutation’s pathogenicity. The comparison of the currently known impact of the detected *ARID1A* and *TP53* mutations (databases) with our *in silico* evaluation and IHC/functional analyses is summarized in Table 3.

**Table 3:** Evaluation of the impact of the detected *TP53* and *ARID1A* mutations based on databases, *in silico* prediction pipeline, immunohistochemistry and functional assay.

Gene	Variant	exon	VAF %	Type	ClinVar	Cosmic	*in silico*^‡^ prediction	IHC^§^	Functional assays	Overall evaluated pathogenicity
**ARID1A**	p.Q583X	3	14	nonsense	novel	novel	NA	10	NA	pathogenic
	p.Q1188X^†^	14	18	nonsense	novel	pathogenic	NA	5	NA	pathogenic
	p.K1230Mfs	14	22	fs	novel	novel	NA	25	NA	pathogenic
	p.P1618S^†^	18	22	missense	novel	pathogenic	benign (5/14)	5	NA	likely pathogenic
	p.R1721X	20	19	nonsense	novel	pathogenic	NA	<1	NA	pathogenic
	*p.E1779G*	20	46	missense	NA	novel	benign (1/14)	80	NA	likely benign
	p.Q1894X	20	61	nonsense	novel	pathogenic	NA	3	NA	pathogenic
	p.V2244G	20	18	missense	novel	pathogenic	benign (6/14)	30	NA	likely pathogenic
**TP53**	*p.P80S*	4	29	missense	VUS	novel	benign (4/14)	wt	wt	likely benign
	p.L194P	6	76	missense	VUS	novel	patho (11/14)	aberrant	deleterious	pathogenic
	p.R196X	6	6	nonsense	pathogenic	pathogenic	NA	wt	NA	pathogenic
	p.R273P	8	62	missense	patho/likely patho	novel	patho (12/14)	aberrant	deleterious	pathogenic
	p.G245D	7	70	missense	patho/likely patho	novel	patho (12/14)	aberrant	deleterious	pathogenic
	p.R280K	8	60	missense	VUS	novel	patho (12/14)	aberrant	deleterious	pathogenic
	c.75–1G>T	i2	19	splice	novel	novel	NA	wt + aberrant clone	NA	likely pathogenic
	c.782 + 1G>C	i7	16	splice	novel	pathogenic	NA	wt	NA	likely pathogenic
	c.919 + 1G>A	i8	60	splice	novel	pathogenic	NA	wt	NA	likely pathogenic

^†^Mutations detected in the same melanoma sample, ^‡^in the brackets is the number of predictors assessing mutation as pathogenic out of the 14 predictors used, final evaluation by *in silico* predictors was considered pathogenic when more than seven predictors suggested pathogenicity of mutation, ^§^evaluation of ARID1A expression shows the percentage of tumor cells with nuclear staining of any intensity, TP53 was evaluated as aberrant or wild-type, fs – frameshift, NA – not evaluated (recorded in the Clinvar database, but the clinical significance is not provided), wt – normal expression pattern or functional behavior compared to wt protein, VAF – variant allele frequency.

**Figure 2:**
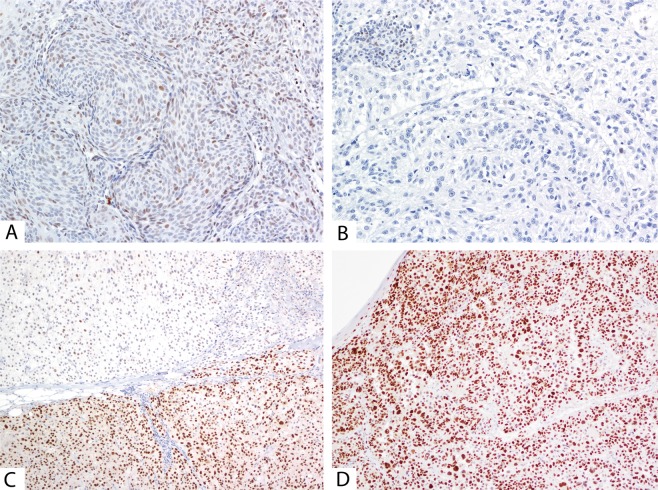
Representative examples for the ARID1A and p53 staining. (**A**) weak and focally strong ARID1A positivity in a case with a novel benign p.(E1779G) missense mutation, 200x magnification; (**B**) absence of ARID1A staining in a melanoma with a novel nonsense pathogenic mutation p.(R1721X), 200×; (**C**) wild-type p53 staining with an aberrant clone with nuclear p53 overexpression in a melanoma with detected novel splice *TP53* mutation c.75-1G>T, affecting the consensus splice site in intron 2, 100×; (**D**) aberrant p53 staining with diffuse strong nuclear expression in a melanoma that developed pathogenic missense *TP53* mutation p.(G245D), 100×.

When comparing the impact of the detected ARID1A mutations, one sample with a missense *ARID1A* p.(E1779G) mutation (which had been previously classified as VUS and predicted as benign using our *in silico* pipeline) showed a strong expression of ARID1A in 80% of the tumor nuclei, which is a similar extent of expression to that found in tissues with wild-type *ARID1A* (Fig. 2A). Interestingly, a different sample with a nonsense mutation p.(R1721X) with VAF 19% showed immunohistochemical expression of ARID1A in <1% of tumor nuclei (Fig. 2B). Moreover, significantly reduced ARID1A expression (the nuclear ARID1A positivity ranged between 1 and 30%) was observed in all cases possessing class 4/5 mutations.

We observed aberrant p53 expression (Fig. 2D) in four of the five melanomas with pathogenic *TP53* mutation. The wild-type p53 expression was observed in one case with a novel variant (predicted as likely benign by *in silico* and functional evaluation), and in two cases with variants affecting the canonical splice-donor sites. However, there was one case with a transversion affecting the canonical splice-acceptor site in which we observed two different types of expression within a single tumor lesion – there was a wild-type p53 expression, found together with a focus of clonal-like aberrant p53 expression (Fig. 2C).

**Figure 3:**
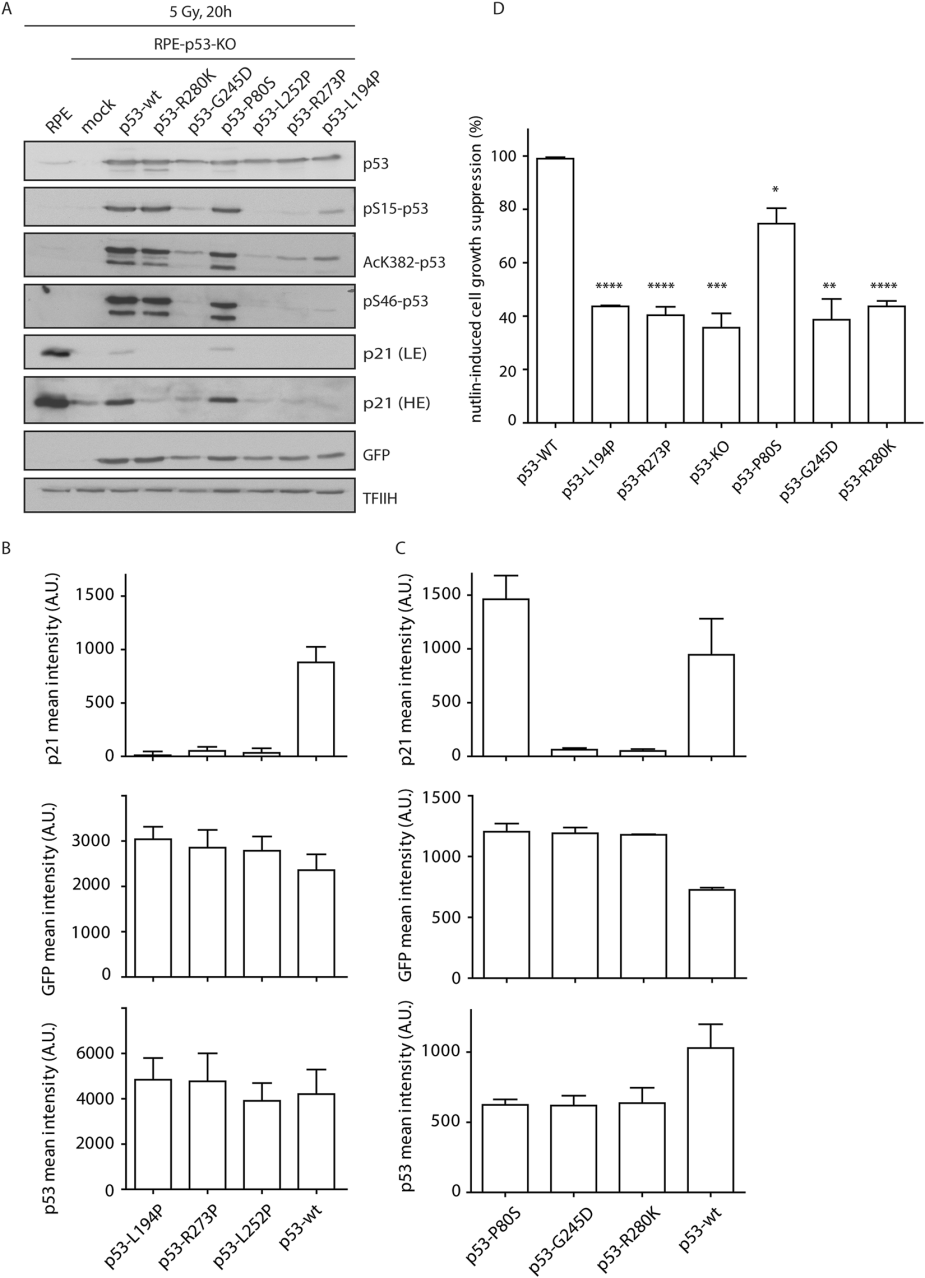
Functional analysis of the detected *TP53* mutations. (**A**) RPE-TP53-KO cells were transiently transfected with a plasmid (expressing either wild-type or mutant p53) and a GFP reporter and were collected 20 hours after exposure to ionizing radiation. Parental RPE cells and mock transfected RPE-TP53-KO cells were used as controls. Cell lysates were probed with indicated antibodies by immunoblotting. All the samples were run in the same experiment, same blotting, on multiple gels. TFIIH was used as a loading control, GFP as a control of transfection. Grouped blots are cropped from different exposures (depending on the antibody’s signal strength), full length blots are included in the Supplementary Information File. (**B,C**) RPE-TP53-KO cells were processed as in (**A**) and analyzed by flow cytometry. A mean intensity of p21 and p53 was determined in the GFP positive cells. (**D**) RPE-TP53-KO cells were stably reconstituted with the wild type or mutant p53 and were grown in the presence of nutlin-3 for 7 days. The level of cell growth suppression was determined by resazurin assay and was normalized to the cells reconstituted with the wild type p53 (n = 3).

The impact of the missense *TP53* mutations on cellular functions was studied by reconstitution assay, in which we expressed either the wild-type or mutant p53 in TP53 knock-out cells (Fig. 3A–C). Immunoblotting and flow cytometry showed that the *TP53* mutation p.(P80S) was able to induce comparable levels of p21 expression as the wild-type p53. Conversely, p53 mutants p.(L194P), p.(R273P), p.(G245D) and p.(R280K) failed to induce p21 and scored similarly as the control loss-of-function *TP53* mutation p.(L252P). In addition, the p.(L194P), p.(R273P), p.(G245D) mutants showed defects in acetylation of K382 and phosphorylation of S15 and S46. The cell growth suppression was only slightly reduced in the p.(P80S) mutant, which is comparable with wild-type p53, whereas p.(L194P), p.(R273P), p.(G245D) and p.(R280K) mutants showed an impaired ability to suppress cell growth, which is comparable with the loss-of function control RPE-p53-KO cells (Fig. 3D).

### Occurrence of mutations in relation to clinico-pathological variables

The relationship between the occurrence of mutations in frequently mutated genes and the age, histological subtype, tumor stage, sex or tumor localization was studied (Table 4). The melanomas with *ARID2* mutation were significantly associated with a higher age at diagnosis, both when the age was dichotomously categorized according to the mean age (χ^2^ = 5.07, p = 0.024) and when age was evaluated as a continuous predictor (logistic regression, Wald statistics = 4.03, p = 0.044). The *PARD3* mutated melanomas were also associated with a higher age at diagnosis, but this result was only marginally significant (χ^2^ = 4.00, p = 0.046). This effect was not significant when age was treated as a continuous variable (logistic regression, Wald statistics = 3.52, p = 0.061). The *ARID1A* mutations were more frequently detected on the head + upper + lower extremities, compared to the trunk (χ2 = 5.10, p = 0.024).

**Table 4:** Statistically significant associations of the frequently mutated genes with clinico-pathological variables.

Genes	ARID1A		*p*-value	ARID2		*p*-value	PARD3		*p*-value
Variables	wt	mut		wt	mut		wt	mut	
total	108	6		106	8		107	7	
**Histological subtype**			0.177			0.564			0.512
NM	42	4		42	4		44	2	
SSM	66	2		64	4		63	5	
**Tumor stage**			0.608			0.094			0.515
pT1 + pT2	26	2		28	0		27	1	
pT3 + pT4	82	4		78	8		80	6	
**Location**			**0.024**			0.167			0.165
Sun non-exposed	68	1		66	3		63	6	
Sun exposed	40	5		40	5		44	1	
**Age**			0.965			**0.024**			**0.046**
≤median	55	3		57	1		57	1	
>median	53	3		49	7		50	6	

Sun exposed location = head, lower- and upper extremities; sun non-exposed location = trunk. P-values are based on Chi-squared tests, all significant p-values are indicated **in bold**. NM – nodular melanoma, SSM - superficial spreading melanoma. The full list of statistical associations of frequently mutated genes with clinic-pathological variables is in Supplementary Table 2.

**Figure 4:**
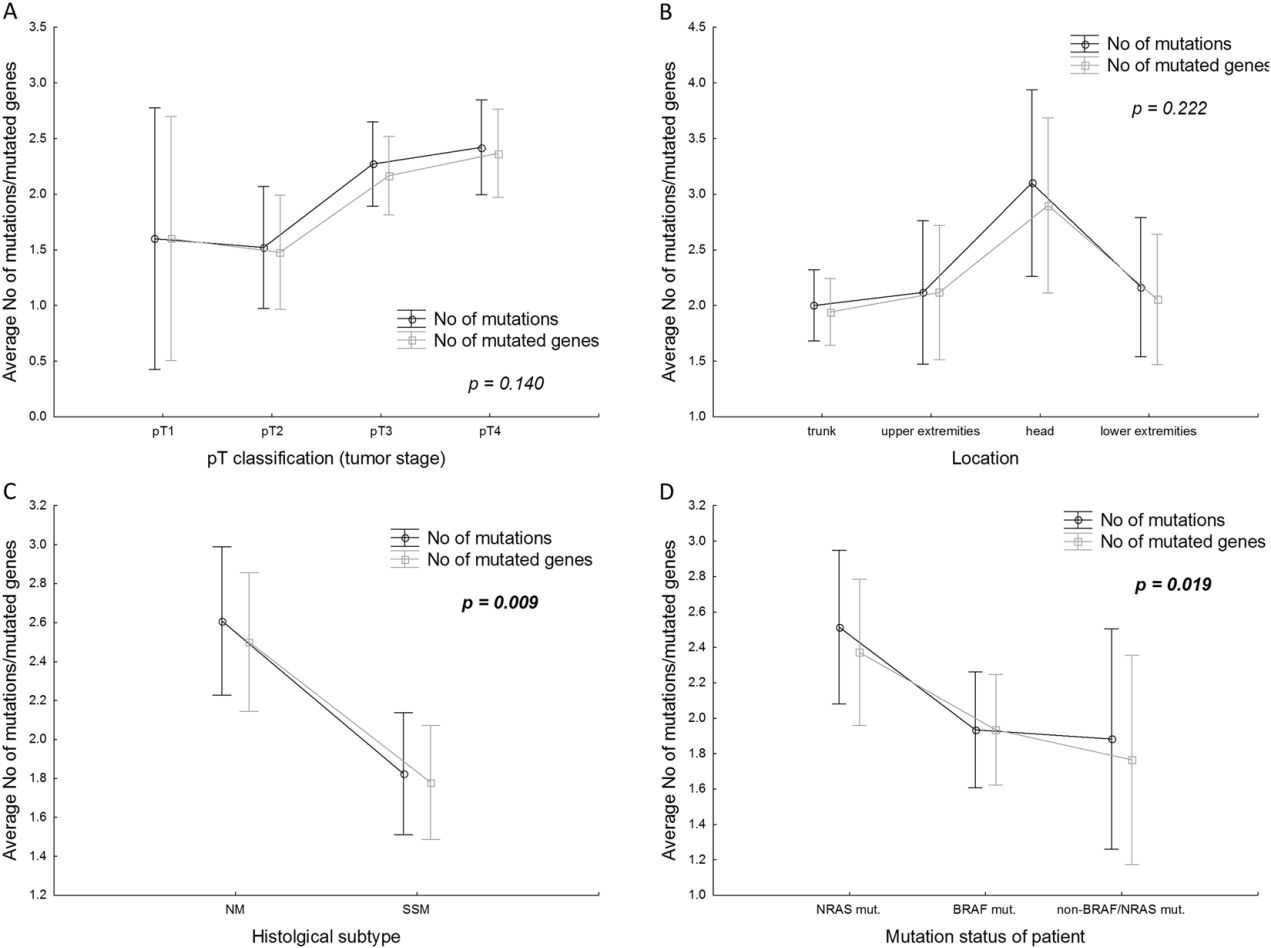
The average number of mutations/mutated genes in relation to the selected clinico-pathological variables in the cohort of 114 patients (**A–C**) and 113 patients (**D** – one case with concurrently detected both *BRAF* and *NRAS* mutations was excluded from this kind of analysis). P-values are based on ANOVA, significant p-values are indicated in bold. Vertical bars denote 0.95 confidence intervals.

Furthermore, we also analyzed the relationship between the number of mutations/mutated genes and the clinico-pathological variables (Fig. 4). The average number of pathogenic, likely pathogenic mutations and/or mutated genes per sample was significantly associated with particular histological subtypes. Both mutations and mutated genes were enriched in NM in comparison with SSM (ANOVA, F (2, 111) = 4.919, p = 0.009; Fig. 4C), and also in *NRAS* mutated patients when compared with *BRAF*^*mut*^ or non-*BRAF*^*mut*^/non*-NRAS*^*mut*^ (ANOVA, F (4, 218) = 3.001, p = 0.019; Fig. 4D). Non-significant correlations were observed for tumor stage and location (Fig. 4A,B).

The most prominent substitutions were the C>T (19%; 6193/32848) and G>A (19%; 6124/32848) transitions. The C>T substitutions constituting 5.7–39.3% (mean 16.8%, median 15.8%) of all the detected mutations with VAF >10% per patient were more common in NM when compared with the SSM cases (ANOVA, F (1,112) = 7.085, p = 0.009). They were also more frequently localized on the head than on other parts of the body (ANOVA, F (3,110) = 5.103, p = 0.002). There were no significant differences in the distribution of C>T substitutions among the tumor stage groups.

### Prognostic value of *BRAF* and *NRAS* mutation status

Nine patients treated by targeted therapy (including BRAF inhibitors, anti-CTLA-4, and anti-PD-1) were excluded from the analyses to minimize the potential beneficial influence of the treatment on the length of survival. Out of those 105 patients 37 patients underwent adjuvant interferon alpha (IFN-α) therapy. There was no significant difference in the survival rates among *BRAF*^*mut*^, *NRAS*^*mut*^ and/or non-*BRAF*^*mut*^/non-*NRAS*^*mut*^ in any of the evaluated outcomes (DFS: χ^2^ = 0.767, p = 0.681, LFS: χ^2^ = 1.239, p = 0.538, MFS: χ^2^ = 2.582, p = 0.275).

## Discussion

Melanoma is a complex and heterogeneous disease with multiple signaling pathways implicated in its pathogenesis. In our cohort of primary NM and SSM, the spectrum of the genes affected by somatic class 4/5 mutations included predominant *BRAF* and *NRAS* mutations which were mutually exclusive, with the exception of one case. However, concomitant *BRAF* and *NRAS* alterations, both located outside the hot-spots *BRAF*^*V600*^/*NRAS*^*Q61*^, were also described in another study^12^. We identified somatic mutations with VAF >3% in the following genes: *ARID1A*, *ARID2*, *ATM*, *BRCA2*, *BRIP1*, *ESR1*, *IDH1*, *KDR*, *MAP2K1*, *MET*, *NBN*, *PARD3*, *PPP6C*, *PTEN*, *SF3B1*, *TJP1*, *TP53* and *ZEB2*. Many of these have already been described in CM^3–5,13,14^. Interestingly, a majority of the detected class 4/5 mutations in *ARID2*, *KDR*, *MET*, *PARD3* and *ZEB2* were novel (Table 2). The *ATM* and *PPP6C* mutations have been previously described in a cohort of sun-exposed melanomas^5,13^. Somatic *BRCA2* mutations are rare events and, additionally, germline mutations in *BRCA2* have also already been described in melanoma in a few studies, although its reported association with an increased risk of melanoma is questionable^15^. Mutations in the *IDH1* gene (codes for isocitrate dehydrogenase 1) have been associated with the development of multiple malignances. Relatively frequent *IDH1* mutations (4.9%) were detected also in metastatic melanoma associated with *NRAS* mutation^16^. We found a co-occurrence of *IDH1* with *NRAS* mutations in 3/5 primary melanomas (Fig. 1). In the *SF3B1* gene we identified six class 4 mutations in 5/114 (4.4%) samples. Somatic *SF3B1* mutations have been described in several malignancies, including mucosal^3^ and uveal melanoma, but not in CM^2,17^.

In 6 patients (6/114, 5.3%) there were no class 4/5 mutations detected in any of the 54 evaluated genes, which suggests the involvement of other genes which were not covered by our sequencing panel, or other alternative molecular mechanisms affecting the genes, such as promoter mutations, copy number variations, translocations or the involvement of the non-coding genome in melanoma pathogenesis^9^.

CM is a heterogeneous disease, where the interplay of several factors, including environmental exposures, genetic and other factors, leads to the formation of melanomas with different biological and clinical characteristics^2,3^. UV radiation has been suggested as a major mutagenic factor in melanoma^4,13,18^. The C>T transitions are typically associated with ultraviolet radiation-induced DNA damage^18–21^. Melanomas frequently show UV signature mutations, but not all melanomas are associated with UV exposure^19^. In our cohort, prominent C>T substitutions accounted for 6–39% (mean 16.8%) of all the mutations per case. None of the cases passed the previously set criteria for UV signature (>60% C>T substitutions)^8^, which suggests that middle-sized target panels (about 200 kb) are not suitable for the assessment of UV signature. Different mutation spectra between melanomas of the skin from locations with a high degree of cumulative sun damage (high-CSD melanomas) and low-CSD melanomas have also been described^19^, including increased frequencies of mutations of *TP53* and *ARID2* in high-CSD melanomas^4^. In our cohort, which is limited in its size, no correlation was found between the frequency of class 4/5 mutations in those genes and the location of the tumor. A higher mutation rate was observed in NM compared to SSM (p = 0.009). The same pattern was also observed by others in the metastatic NM cohort when compared with SSM^22^. The mutation rate in melanomas occurring on chronically sun exposed skin was found to be approximately five times higher than on skin without chronic sun exposure^8,23^. In our study, in which we only considered class 4/5 mutations, there was a clear trend where more mutations were detected in the melanomas located on the head (Fig. 4B). Nevertheless, the correlations are limited as only 10 cases were considered (5 NM and 5 SSM). The results are also influenced by the relatively small size of our panel, which does not allow for the assessment of the exact number of mutations or tumor mutation burden.

Germline, likely pathogenic mutations were identified in *ATM* c.8228C>T, p.(T2743M), *MLH3* c.958T>G, p.(C320G), and *IDH1* c.245G>A, p.(R82K) in three independent patients. The *ATM* gene (coding for apical double strand break repair^24^ kinase) was shown to be a rare susceptibility gene in familial melanoma^25^. However, the germline *MLH3* (involved in mismatch repair - MMR) mutation does not seem to be involved in tumorigenesis, considering the high age at diagnosis and microsatellite stable phenotype of the affected tumors. It was shown that MMR complex MutLγ, which is formed by the MLH3 and MLH1 protein, is a less efficient MMR complex than MutLα. That demonstrates that the MLH3 mutations alone do not interfere with MMR^26^. The clinical impact of a novel, likely pathogenic germline *IDH1* mutation p.(R82K) is unclear.

It has been described that most melanomas possess potentially actionable mutations in the components of the MAPK and PI3K-AKT pathways, cell cycle regulation and chromatin remodeling^3,4,7,8,27,28^. In this study, the majority of identified mutations affected genes coding for components of the RAS signaling, including mitogen-activated protein kinase (MAPK) or phosphatidylinositol 3-kinase (PI3K)-AKT/PKB signaling pathway. Other recurrently altered intracellular signaling pathways in our patients included DNA damage response, cell cycle regulation, and chromatin remodeling. There were also several genes frequently affected by somatic mutations, such as the genes coding nuclear hormone receptor (*ESR1*), RAS signaling pathway members (*MET*, *KDR*), DNA repair genes (*BRIP1*, and *NBN*), and genes coding for proteins involved in tight junction assembly or regulation of epithelial-to mesenchymal transition (*PARD3*, *TJP1*, and *ZEB2*), which have not yet been described as significantly mutated genes in primary CM. The TJP1 and PARD3 protein have not been described to play a role in melanomagenesis at all so far. The epithelial-to-mesenchymal transition transcription factors ZEB2 and SNAI2 have been shown to control melanocyte differentiation^29,30^. Loss of ZEB2 expression was already reported to be associated with poor melanoma-specific survival^30^.

The *in silico* prediction of the pathogenicity of the *TP53* and *ARID1A* mutations was compared with the protein expression and function. Pathogenic mutations showed aberrant protein expression by immunohistochemical analysis and/or impaired function, as confirmed by the functional assay developed in our study. All nonsense *ARID1A* mutations showed a loss of ARID1A expression by IHC. Interestingly, one case with a *TP53* nonsense mutation (VAF <10%) and two *TP53* variants affecting the consensus splice-acceptor site showed a wild-type pattern of p53 expression when evaluated by IHC (Table 3). The wild-type expression of p53 in those cases could be explained by the low frequency of nonsense *TP53* mutations present (suggesting limited impact of variants detected with low frequency) and/or by nonsense-mediated RNA decay in case of all variants leading to premature terminal codon^31^. All the detected *TP53* mutations (except for the one splice mutation c.75–1G>A) have already been described in the IARC TP53 database (somatic mutations - release R19, July 2018;^32^), and are considered by *in silico* prediction to be pathogenic (except the benign variant p.P80S). We developed cell/based functional assays, and we identified that p.(L194P), p.(R273P), p.(G245D) and p.(R280K) are loss-of-function mutations of the *TP53*.

The consistent results obtained by different approaches support the accuracy of the used *in silico* analysis algorithm. However, we did observe conflicting impact interpretation results when comparing the classification in databases, the *in silico* predictions and literature in case of the *CDK4*. Its mutation p.(Arg24His), rs104894340 was predicted by our *in silico* prediction pipeline to be likely benign, however, in the ClinVar database this variant is classified as likely pathogenic somatic, albeit without strong assertion criteria. The same variant was also detected in a large Norwegian family with multiple atypical nevi and malignant melanomas^33^. Another mutation in the same codon p.(Arg24Cys), rs11547328 was classified as class 4/5 in the ClinVar. Therefore, we concluded that this variant is likely pathogenic. Similarly, the *BRCA2* mutation p.(P2532L) was evaluated in the ClinVar database as likely benign by a single submitter, with its clinical significance being unknown according to the BIC and BRCA Exchange databases. However, the Cosmic database (including only FATHMM prediction), and our *in silico* algorithm suggested it to be pathogenic, and therefore we evaluated this variant as likely pathogenic. The pathogenicity of this variant was also supported by Align-GVGD^34^.

Several studies have identified clinical associations with *BRAF*^*V600*^ mutations, including melanoma subtype, primary tumor location, and prognosis^35–38^. Another study observed a higher rate of *NRAS* mutations in NM compared to SSM^22^. On the contrary, we did not find any significant correlations of the frequently mutated genes, including *BRAF*^*mut*^ or *NRAS*^*mut*^ with the histological subtype (NM versus SSM), stage, location of the primary melanoma or the outcome. Nevertheless, our analysis suggested a significant association of the *ARID2* or *PARD3* mutations with a higher age at diagnosis (p < 0.05), and the loss-of-function *ARID1A* mutations were associated with sun exposed skin (p = 0.024). We did not observe any significant correlations of the frequent mutations with any of the studied outcomes (DFS, LFS or MFS). The strongest prognostic marker remains Breslow thickness of the primary melanoma and SLN positivity^39^. Considering other morphological parameters, the melanization of metastatic melanoma also seems to be an important prognostic and predictive marker^40,41^.

Modern targeted treatment strategies (including BRAF inhibitors and immunotherapy by immune checkpoint inhibitors) are currently widely used^42,43^. New potential therapy targets, such as inhibitors of CDK4/6, MDM2/p53, c-KIT, PI3K/Akt/mTOR, ERK, or IDH1, have also been considered for melanoma treatment^27,28,44^. The mutations *BRAF*^*V600*^ and *NRAS*^*Q61*^ account for a large proportion of actionable mutations in CM and represent logical targets for downstream (MEK) inhibitors^4,13,37,45,46^. In this study we demonstrated relatively common pathogenic mutations in the *KDR* and *MET* genes, which code for tyrosine kinase growth factor receptors VEGFR2 and HGFR, respectively. Both these genes activate several signaling cascades, including the MAPK, RAS-ERK or PIK3-AKT signaling pathway. The *KDR* and *MET* mutations could represent new therapeutic options. The MET is currently studied as a potentially druggable target in non-small cell lung cancer^47^.

### Limitations

Several genes were not covered by our analysis due to the high stringency of the designed probes, and therefore the mutation analysis does not cover some of the already known rare (e.g. *AKT1*^2,3^ and *SMARCA4*^4^) or frequently mutated genes (e.g. *MAP2K2*^14^) in melanoma. From a clinical point of view, it would certainly be interesting to explore the correlations between TIL and tumor mutation burden (TMB). Unfortunately, the evaluation of TMB was impossible due to the small size of the sequenced targets (about 2Kbp) and the fact that the sample set is limited.

## Conclusion

To the best of our knowledge, our analysis is the first to include the determination of pathogenicity of the detected mutations using biostatistical analyses and immunohistochemical and/or functional assays. We describe the spectrum and prevalence of class 4/5 mutations in primary NM and SSM, including several novel variants. Additionally, the functional analysis identified two loss-of-function *TP53* missense mutations, which were previously classified as VUS. As well as the commonly described RAS-, cell cycle regulation- and chromatin remodeling pathways in CM, we revealed frequently mutated genes coding for proteins involved also in DNA damage response and cellular tight junction regulation. The newly identified significantly mutated genes included *ATM*, *KDR*, *MET*, *PARD3*, *SF3B1* and *ZEB2*, which could all be of clinical importance. Our results suggest that with the development of new therapeutic possibilities, not only *BRAF* testing but complex molecular testing of CM may become an integral part of the decision process concerning the treatment of patients with melanoma.

## Material and Methods

### Patients and samples

Formalin-fixed paraffin-embedded (FFPE) tissue blocks of primary melanomas collected between the years 2006–2017 were obtained from the archive files processed at the Institute of Pathology and the Department of Dermatology and Venereology, First Faculty of Medicine, Charles University and General University Hospital in Prague. A review of the hematoxylin and eosin stained slides was performed in all cases and areas of non-tumor and tumor tissues were marked for macrodissection (with estimation of tumor cell percentage in the selected area; ranging between 60–99%). For this project, the DNA was isolated from more than 200 samples, but only about 50% of those passed the qualitative and quantitative requirements for NGS (massive parallel sequencing) analysis and biostatistical evaluation. The clinical and pathological characteristics of the 114 successfully analyzed patients and tumors are summarized in Table 1. The available patient information with respect to the follow-up data was updated at the end of this project in April 2019. The mean age of patients at diagnosis was 62 years (median 66, range 24–93 years). The melanoma samples were divided according to the TNM classification (7^th^ Edition) into four groups (pT1 ≤1 mm, pT2 >1 and ≤2 mm, pT3 >2 and ≤4 mm, and pT4 >4 mm). Tumor infiltrating lymphocytes (TILs) were evaluated within our previous study^48^ which included a subset of samples analyzed also in this present study. In our sample set, mainly melanomas with a larger thickness of the primary tumor were selected in order to get enough material for DNA isolation. A corresponding non-tumor tissue from the selected patients was used for DNA isolation to determine germline or somatic origin of the identified mutations.

### Ethics statement

In compliance with the Helsinki Declaration, the project has been approved by The Ethics Committee of General University Hospital in Prague (ethical approval number č.j. 56/15 Grant VES 2016 AZV 1. LFUK). The ethics committee which approved this study waived the need for informed consent. The methods were carried out according to the approved ethical guidelines.

### DNA isolation and quality control

DNA from formalin-fixed paraffin-embedded (FFPE) tissue blocks was isolated using standard procedures implementing cobas® DNA Sample Preparation kit (Roche; Germany) or an automated MagCore® nucleic Acid Extractor using MagCore Genomic DNA FFPE One-step kit, (RBC Bioscience). Every sample underwent a quality control test of amplification efficacy by qPCR (5 ng DNA of sample was amplified using 5x HOT FIREPol® EvaGreen® HRM Mix NO ROX (Solis Biodyne), only the samples which passed the quality criteria (C_t_ <35 for a 180 bp product amplification) were used for the library preparation.

### NGS analysis and biostatistical processing of data

Samples for sequence capture NGS were prepared using the KAPA HyperPlus kit according to Seq Cap EZ protocol (Roche NimbleGen). The target sequences were enriched using a panel of hybridization probes against multiple targets (72 genes or gene parts; 219 kbp; NimbleGen, Roche). High stringent probes were designed to match only one target to avoid the enrichment of homologous sequences. The prepared sample libraries were pair-end sequenced by MiSeq instrument (Illumina) using MiSeq Reagent Kit v3 (150 cycles; Illumina). Initial data sorting and demultiplexing to fastq files was carried out with the MiSeq software. NextGENe software (Softgenetics, State College, PA) was used for main biostatistical analysis of the sequencing data. PCR duplicate reads were removed using NextGENe Sequence Operation tool. The remaining unique paired reads in fastq file format were converted to fasta file format using NextGENe Format Conversion tool with low quality reads removal (median score <25; number of uncalled bases ≥3; called bases number <40, reads trimmed or rejected when ≥3 bases with score ≤20). The remaining unique high-quality paired reads in fasta format were mapped on human GRCh37 genome version by NextGENe using Sequence Alignment tool (allowable mismatched bases – 0; allowable ambiguous alignments – 10; seeds – 21 bases, move step 1 base, allowable alignments – 80; overall matching base percentage ≥95%) with a mutation filter VAF ≥5%. The alignment results were exported as a Mutation report (txt file), and the mapped reads were exported in a BAM format. The nonsynonymous variants in exons and splice variants in adjacent intronic regions with minimal average coverage 100x and VAF >10% were evaluated, and manually inspected in a BAM file using an IGV viewer (Broad Institute) in order to avoid false positive variations. The copy number variations (CNVs) were not evaluated due to the limited quality of the FFPE samples. A majority of the samples had significantly fragmented DNA which leads to non-homogenous length of the reads and coverage of target areas in the NGS panel.

### Variant prioritization

Variants of each sample included in the Mutation Report obtained from the NextGENe software NGS data analysis were prioritized as follows: (i) all pathogenic and likely pathogenic variants based on Clinical Significance from ClinVar database, (ii) all nonsense, frameshift, in-frame, no-start and no-stop variants, and (iii) all splice variants in consensus splice sites. In the next step, (iv) all missense variants with a population frequency ≤0.01 and with a non-evaluated frequency based on dbNSFP 1000G_AF, and (v) potential splice variants with a rank score ≥0.5 based on dbNSFP_dbscSNV database were prioritized.

### Databases and *in silico* prediction pipeline

In order to assess the impact of the prioritized variants we searched several databases and/or performed comprehensive analysis by 14 different *in silico* prediction tools implemented in the Variant Effect Predictor (VEP, Ensembl, http://grch37.ensembl.org/Homo_sapiens/Tools/VEP/)^49^. In VEP, the predictors were selected based on (i) evaluation of amino-acid change and protein structure (SIFT, PolyPhen-2, FATHM, PROVEAN and VEST3), (ii) evolutionary conservation (GERP++, MutationAssessor and SiPhy), (iii) evaluation of multiple databases information (CADD, MutationTaster and LRT) and (iv) based on combined algorithms (M-CAP, MetaLR, MetaSVM). In all the selected predictors, the rankscore which ranged from 0 (=likely benign) to 1 (=likely pathogenic), was evaluated. We considered a variant as “likely pathogenic” if at least 7/14 of selected predictors scored the variant as ≥0.8. The remainder of the variants was considered as likely benign. (Supplementary Table 1).

### Variant characterization

Somatic mutations which were not found in the literature, the Single Nucleotide Polymorphism Database (dbSNP, http://www.ncbi.nlm.nih.gov/SNP/), the ClinVar Database (https://www.ncbi.nlm.nih.gov/clinvar/), or in the Catalogue of Somatic Mutations in Cancer (COSMIC, http://www.sanger.ac.uk/cosmic; databases accessed March 28, 2019) were considered as novel. Gene-specific databases were searched in the case of *TP53* (IARC TP53 Database, http://p53.iarc.fr/), *BRCA1* and *BRCA2* (the BIC database, https://research.nhgri.nih.gov/bic/ and BRCA exchange, https://brcaexchange.org).

The protein truncating mutations, including nonsense, frameshift and any type of variants classified pathogenic in the ClinVar database were considered as “pathogenic”, also referred to as the class 5. The category of “likely pathogenic” mutations, also referred to as the class 4 mutations, included splice mutations in cryptic splice sites and mutations which are either (i) currently classified as Variants of Unknown Significance (VUS, i.e. conflict of interpretations of pathogenicity, uncertain significance, or clinical significance not provided by the ClinVar database) or (ii) novel variants (described neither in the ClinVar, nor in the COSMIC) that were suggested to be pathogenic by our *in silico* prediction pipeline, as is described above.

All variants classified as benign in the mutation databases (ClinVar and/or COSMIC) or novel and VUS variants suggested to be benign by our *in silico* prediction pipeline (described above) were considered as “benign”. These benign variants were excluded from further statistical analyses.

### Commonly mutated pathways

A pathway was considered as altered if at least one gene in the pathway contained pathogenic or likely pathogenic somatic mutation. The association of proteins with functional pathways was judged according to the information obtained from the largely used database Gene Cards (www.genecards.org), including information from e.g.: Kyoto Encyclopedia of Genes and Genomes (KEGG), NCBI databases, CIViC, GeneCards, UniProtKB/Swiss-Prot, Tocris, Reactome, STRING interaction database v11.0^50^, and/or literature.

### Functional assay

The ionizing radiation-induced expression of p21 (which is an established TP53 target), defects in acetylation of K382 and phosphorylation of S15 and S46 (which are posttranslational modifications needed for p53 to function^51^) were analyzed by western blot and flow cytometry. Suppression of cell proliferation in response to treatment with an mdm2 inhibitor nutlin-3^52^ was also studied by a proliferation assay.

#### Cell lines

Human hTERT-RPE-1 cells (referred to as RPE cells; ATCC) were grown at 37 °C and 5% CO_2_ in DMEM supplemented with 6% FBS (Gibco), glutamine (2 mM), penicillin (100 U/ml), and streptomycin (100 g/ml). All cell lines were regularly checked for the absence of mycoplasma infection using MycoAlert Plus reagent (Lonza).

To generate RPE-TP53-KO cells, human hTERT-RPE-1 cells were transfected with a p53-CRISPR/Cas9 KO Plasmid (Santa Cruz, sc-416469) and p53-HDR Plasmid (1:1) and selected by puromycin (10 μg/ml) for 3 weeks^53^. The integration of the HDR cassette into the *TP53* locus was confirmed by sequencing and loss of p53 expression by immunoblotting. Transiently expressed wild-type or mutant *TP53* were cloned into pIRES2-EGFP-TP53 plasmid allowing gating for p53 expressing cells according to the EGFP signal. The pIRES2-EGFP-p53-WT plasmid (Addgene plasmid # 49242) and IRES2-EGFP reporter were gifted to us by Dylan Taatjes. The site-directed mutagenesis of individual variants was performed by Gibson assembly kit (NEB) of two PCR fragments into the NheI/BstxI sites of the pIRES2-EGFP-p53-WT plasmid. All plasmids were verified by Sanger sequencing. Plasmid DNA was transfected using Polyethylenimine HCl MAX 40 kDa (Palysciences) in ratio 6:1 to DNA and growth media were changed after 3 hours. Stable cell lines expressing pIRES2-EGFP-p53-WT or *TP53* variants were selected with geneticin (Sigma) for 3 weeks.

#### Antibodies

used in the western blotting or flow cytometry experiments: p53 (sc-6243), TFIIH p89 (sc-293), and p21 (sc-397; Santa Cruz Biotechnology); Phospho-Ser15-p53 (#9284), Phospho-Ser45-53 (#2521), and Acetyl-Lys382-p53 (#2525; Cell Signaling Technology); HRP-conjugated secondary antibodies (Bio-Rad), and Alexa Fluor-labelled secondary antibodies (Life Technologies).

#### Flow cytometry

For the reconstitution of p53 expression in RPE-TP53-KO cells, the transiently transfected cells with pIRES2-EGFP-p53 WT or variant plasmid were seeded and after 48 hours fixated with 4% paraformaldehyde. Cells stained with antibodies against p53, p21, and DAPI were sorted by flow cytometer LSRII (BD Biosciences) and analyzed by the FlowJo software (FlowJo). The mean signal intensity of p53 or p21 per cell was determined in GFP positive cells. Signal intensity in GFP negative cells was subtracted as a background. At least 10000 cells were measured in three independent experiments.

#### Cell proliferation assays

The RPE-TP53-KO cells, stably reconstituted with pIRES2-EGFP-p53 WT or its variants, were seeded in 96-well plates (500 cells/well) and cultivated in the presence of nutlin-3 (0.5 μM, MedChem Express) for 7 days. Resazurin (30 μg/mL) was added to the growth media and the resulting fluorescence signal (Ex = 560 nm, Em = 590 nm) was measured after 1 h using the EnVision plate reader (PerkinElmer), as previously described elsewhere^54^. The level of cell growth suppression induced by nutlin was normalized to the wild-type p53. Samples were measured in hexaplicates in three independent experiments. The bars indicate standard deviation. Any statistical significance was determined by two-sided t-test using Prism software.

### Immunohistochemical analysis

Immunohistochemical analysis was performed manually on 4 µm sections of FFPE tissue, using the avidin-biotin complex method with an antibody against the ARID1A (clone PSG3, dilution 1:1000; Santa Cruz Biotechnology, California, USA) and p53 (clone BP 53–12, dilution 1:200, Zytomed Systems, Berlin, Germany) antigens. Following the pretreatment in 0.01 M citrate buffer (pH 6.0) for ARID1A or 0.01 M citrate buffer (pH 9.0) for p53, the antigen retrieval was performed for 40 min in a water bath at 98°C. The expression of p53 and ARID1A was double-blindly evaluated by two pathologists. Discrepant cases were reviewed and a consensus score was assessed.

The ARID1A expression was scored as a percentage of tumor cells with nuclear staining of any intensity. Cases with less than 1% tumor cells with ARID1A expression were considered as negative.

The p53 protein expression was assessed as “aberrant” (defined as diffuse moderate to strong nuclear positivity of more than 50% of tumor cells or totally negative staining with a positive internal control in non-tumorous tissue) or “wild-type” (defined as low variable staining in less than 50% of the tumor nuclei)^55^.

### Sanger sequencing and microsatellite instability analyses

Analyses were performed as previously described^56^. Variants detected by NGS with VAF >10% (range 11–90%) were selected for confirmation by direct Sanger sequencing. Where available, the corresponding non-tumor tissue of selected cases with detected class 4/5 mutation with VAF 45–55% was analyzed, to determine germline or somatic origin of the selected mutations. The specific designed primers are available upon reasonable request.

### Statistical analyses

The software Statistica (StaSoft, Tulsa, OK) was used to perform the statistical analyses. Associations of mutations with clinico-pathological characteristics were evaluated using either the chi-squared test or logistic regression. The correlation between the number of mutations/mutated genes and the rates of C > T substitutions with clinico-pathological variables was evaluated using ANOVA. Time-to-event analysis was performed with three outcomes – disease-free survival (DFS: death from melanoma was considered as a failure), local recurrence-free survival (LFS: the period from primary diagnosis till the first local recurrence), and distant metastasis-free survival (MFS: the period from primary diagnosis till the first distant metastasis). These analyses included 105 patients without targeted therapy (including immunotherapy by check-point inhibitors). The date of the diagnosis was the date of the primary sample accession in the pathology database. The initial univariate analysis was performed using the log-rank test to determine the differences between cohorts and to construct the Kaplan-Meier estimated survival curves. Cox’s Proportional Hazard Ratio Models were used for univariate and multivariate analysis of risk factors for disease-free survival. The minimal adequate model was achieved through backward elimination of non-significant effects (p > 0.05). All of the performed tests were two-sided and a p-value <0.05 was considered as significant.

## Supplementary information

Supplementary Information

Supplementary Table 1

## Data Availability

The source data are included in this article and its Supplementary Information File or are available from the corresponding author upon reasonable request.
