# Supplementary material for: A comprehensive evaluation of pathogenic mutations in primary cutaneous melanomas, including the identification of novel loss-of-function variants
Source: Sci Rep. 2019 Nov 19;9:17050. doi: 10.1038/s41598-019-53636-x (PMC6863855; doi:10.1038/s41598-019-53636-x)
Supplement: Supplementary file 1 — Supplementary Information [file 41598_2019_53636_MOESM1_ESM.pdf]

**Supplementary Information**

# A comprehensive evaluation of pathogenic mutations in primary cutaneous melanomas, including the identification of novel loss-of-function variants.

Running title: The spectrum and prevalence of mutations in primary melanoma

Ivana Ticha[1*], Jan Hojny[1], Romana Michalkova[1], Ondrej Kodet[2,3,4], Eva Krkavcova[1], Nikola Hajkova[1], Kristyna Nemejcova[1], Michaela Bartu[1], Radek Jaksa[1], Miroslav Dura[1,2], Madiha Kanwal[5], Andra S. Martinikova[5], Libor Macurek[5], Petra Zemankova[6], Zdenek Kleibl[6] and Pavel Dundr[1]

[1]Institute of Pathology, First Faculty of Medicine, Charles University and General University Hospital in Prague, Czech Republic
[2]Department of Dermatology and Venereology, First Faculty of Medicine, Charles University and General Hospital in Prague, Prague, Czech Republic
[3]Institute of Anatomy, First Faculty of Medicine, Charles University, Prague, Czech Republic
[4]BIOCEV, Charles University, First Faculty of Medicine, Vestec Czech Republic
[5]Cancer Cell Biology, Institute of Molecular Genetics, ASCR, Prague, Czech Republic
[6]Institute of Biochemistry and Experimental Oncology, First Faculty of Medicine, Charles University, Prague, Czech Republic

ivana.ticha@vfn.cz
*Correspondence:
Ivana Ticha, Institute of Pathology, First Faculty of Medicine, Charles University and General University Hospital in Prague, Studnickova 2, 12800, Prague 2, Czech Republic

**Supplementary Data.** The panel of genes or gene parts.

AKT1, **AKT3**, ***ARID1A***, **ARID2**, **ATM**, BAP1, **BARD1**, promBIRC5, **BRAF**, **BRCA1**, **BRCA2**, **BRIP1**, **CCND2**, CCND3, ***CDH1***, **CDK4**, ***CDKN2A***, **CYP19A1**, ERBB2, **ERCC3**, **ESR1**, **ESR2**, **F11R**, FOXL2, GNA11, GNAQ, **HNF1B**, HRAS, **IDH1**, **JAM2**, **JAM3**, **KDR**, **KIT**, **KRAS**, *MAP2K1*, MAP2K2, MAPK3, **MDM2**, **MET**, **MITF**, **MLH1**, **MLH3**, **MSH2**, **MSH6**, **MYC**, **NBN**, **NRAS**, **PALB2**, **PARD3**, **PDGFRA**, **PIK3CA**, POLE, **POT1**, **PPM1D**, **PPP6C**, ***PTEN***, **RAD51C**, **RAD51D**, **RB1**, **SF3B1**, SMARCA4, **SMARCB1**, SNAI1, **SNAI2**, SNAI3, promTERT, ***TJP1***, **TP53**, TWIST1, TWIST2, **ZEB1**, **ZEB2**

Genes evaluated in 114 primary cutaneous melanomas are **in bold.** Those with identified class 4/5 mutation are **underlined**. Genes which were only partially evaluated because of low coverage in parts of the genes are ***in italics***.

Several genes did not pass the quality criteria for evaluation due to the stringency of the designed probes (see Methods).

**Supplementary Table 1.** The *in silico* assessment of the significance of prioritized variants.

Included as a separate Excel document

**Supplementary Table 2.** Statistical association of frequently mutated genes with clinico-pathological variables.

| Variables | BRAF | | *p* | Variables | mutation status | | *p* | Variables | mutation status | | *p* |
|---|---|---|---|---|---|---|---|---|---|---|---|
| | wt | mut | | | wt | mut | | | wt | mut | |
| BRAF | 52 | 62 | | NRAS | 79 | 35 | | TP53 | 106 | 8 | |
| **Histological subtype** | | | 0.995 | **Histological subtype** | | | 0.437 | **Histological subtype** | | | 0.185 |
| NM | 21 | 25 | | NM | 30 | 16 | | NM | 41 | 5 | |
| SSM | 31 | 37 | | SSM | 49 | 19 | | SSM | 65 | 3 | |
| **Tumor stage** | | | 0.736 | **Tumor stage** | | | 0.221 | **Tumor stage** | | | 0.976 |
| pT1+pT2 | 12 | 16 | | pT1+pT2 | 22 | 6 | | pT1+pT2 | 26 | 2 | |
| pT3+pT4 | 40 | 46 | | pT3+pT4 | 57 | 29 | | pT3+pT4 | 80 | 6 | |
| **Location** | | | 0.181 | **Location** | | | 0.186 | **Location** | | | 0.385 |
| Sun non-exposed | 28 | 41 | | Sun non-exposed | 51 | 18 | | Sun non-exposed | 63 | 6 | |
| Sun exposed | 24 | 21 | | Sun exposed | 28 | 17 | | Sun exposed | 43 | 2 | |
| **Age** | | | 0.168 | **Age** | | | 0.122 | **Age** | | | 0.129 |
| ≤ median | 23 | 36 | | ≤ median | 44 | 14 | | ≤ median | 56 | 2 | |
| > median | 29 | 27 | | > median | 35 | 21 | | > median | 50 | 6 | |
| ARID2 | 106 | 8 | | MAP2K1 | 106 | 8 | | PARD3 | 107 | 7 | |
| **Histological subtype** | | | 0.564 | **Histological subtype** | | | 0.185 | **Histological subtype** | | | 0.512 |
| NM | 42 | 4 | | NM | 41 | 5 | | NM | 44 | 2 | |
| SSM | 64 | 4 | | SSM | 65 | 3 | | SSM | 63 | 5 | |
| **Tumor stage** | | | | **Tumor stage** | | | 0.976 | **Tumor stage** | | | 0.515 |
| pT1+pT2 | 28 | 0 | | pT1+pT2 | 26 | 2 | | pT1+pT2 | 27 | 1 | |
| pT3+pT4 | 78 | 8 | | pT3+pT4 | 80 | 6 | | pT3+pT4 | 80 | 6 | |
| **Location** | | | 0.167 | **Location** | | | 0.906 | **Location** | | | 0.165 |
| Sun non-exposed | 66 | 3 | | Sun non-exposed | 64 | 5 | | Sun non-exposed | 63 | 6 | |
| Sun exposed | 40 | 5 | | Sun exposed | 42 | 3 | | Sun exposed | 44 | 1 | |
| **Age** | | | **0.024** | **Age** | | | 0.959 | **Age** | | | **0.046** |
| ≤ median | 57 | 1 | | ≤ median | 54 | 4 | | ≤ median | 57 | 1 | |
| > median | 49 | 7 | | > median | 52 | 4 | | > median | 50 | 6 | |
| ATM | 107 | 7 | | KDR | 107 | 7 | | ARID1A | 108 | 6 | |
| **Histological subtype** | | | 0.349 | **Histological subtype** | | | 0.349 | **Histological subtype** | | | 0.177 |
| NM | 42 | 4 | | NM | 42 | 4 | | NM | 42 | 4 | |
| SSM | 65 | 3 | | SSM | 65 | 3 | | SSM | 66 | 2 | |
| **Tumor stage** | | | 0.799 | **Tumor stage** | | | | **Tumor stage** | | | 0.608 |
| pT1+pT2 | 26 | 2 | | pT1+pT2 | 27 | 1 | 0.514 | pT1+pT2 | 26 | 2 | |
| pT3+pT4 | 81 | 5 | | pT3+pT4 | 80 | 6 | | pT3+pT4 | 82 | 4 | |
| **Location** | | | 0.074 | **Location** | | | | **Location** | | | **0.024** |
| Sun non-exposed | 67 | 2 | | Sun non-exposed | 66 | 3 | 0.324 | Sun non-exposed | 68 | 1 | |
| Sun exposed | 40 | 5 | | Sun exposed | 41 | 4 | | Sun exposed | 40 | 5 | |
| **Age** | | | 0.661 | **Age** | | | 0.661 | **Age** | | | 0.965 |
| ≤ median | 55 | 3 | | ≤ median | 55 | 3 | | ≤ median | 55 | 3 | |
| > median | 52 | 4 | | > median | 52 | 4 | | > median | 53 | 3 | |
| MET | 108 | 6 | | | | | | | | | |
| **Histological subtype** | | | 0.621 | | | | | | | | |
| NM | 43 | 3 | | | | | | | | | |
| SSM | 65 | 3 | | | | | | | | | |
| **Tumor stage** | | | 0.151 | | | | | | | | |
| pT1+pT2 | 28 | 0 | | | | | | | | | |
| pT3+pT4 | 80 | 6 | | | | | | | | | |
| | | | 0.752 | | | | | | | | |
| | 65 | 4 | | | | | | | | | |
| Sun exposed | 43 | 2 | | | | | | | | | |
| **Age** | | | 0.965 | | | | | | | | |
| | 55 | 3 | | | | | | | | | |
| > median | 53 | 3 | | | | | | | | | |

Sun exposed location = head, lower- and upper extremities; sun non-exposed location = trunk. *P-values* are based on Chi-squared tests, all significant p-values are indicated **in bold**. NM – nodular melanoma, SSM - superficial spreading melanoma

**Supplementary Figure 1.** Mutations detected by NGS in primary melanomas confirmed from DNA isolated from the corresponding non-tumor tissue.

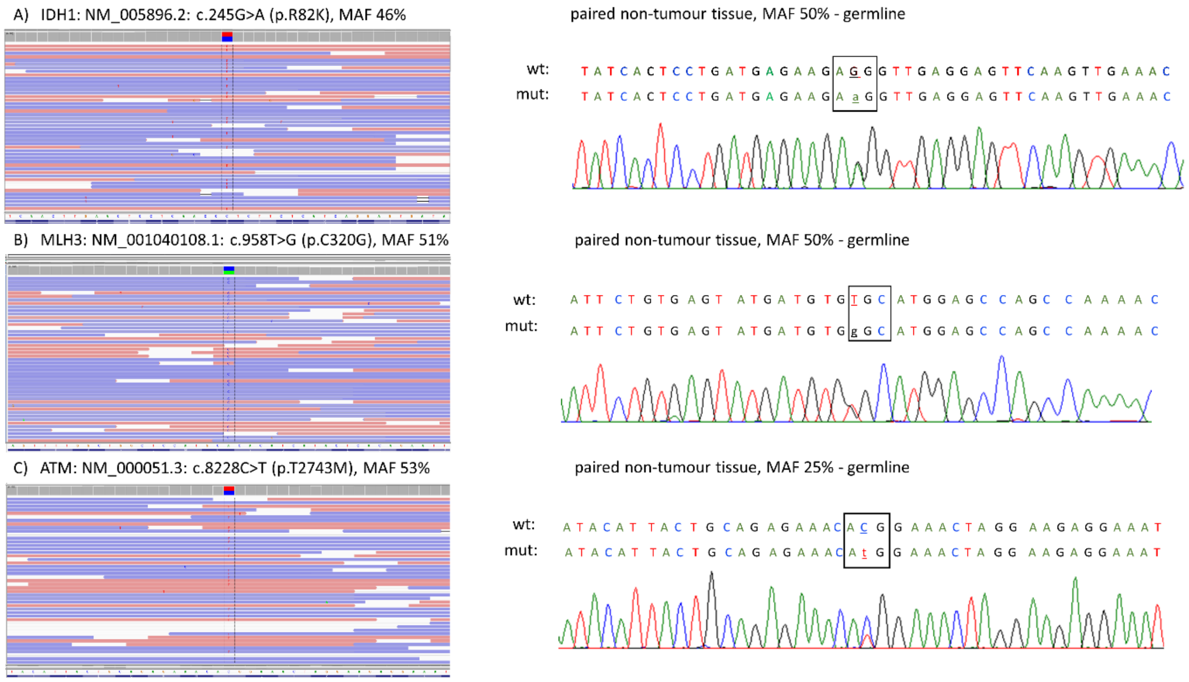

**Extended data - Figure 3A.**

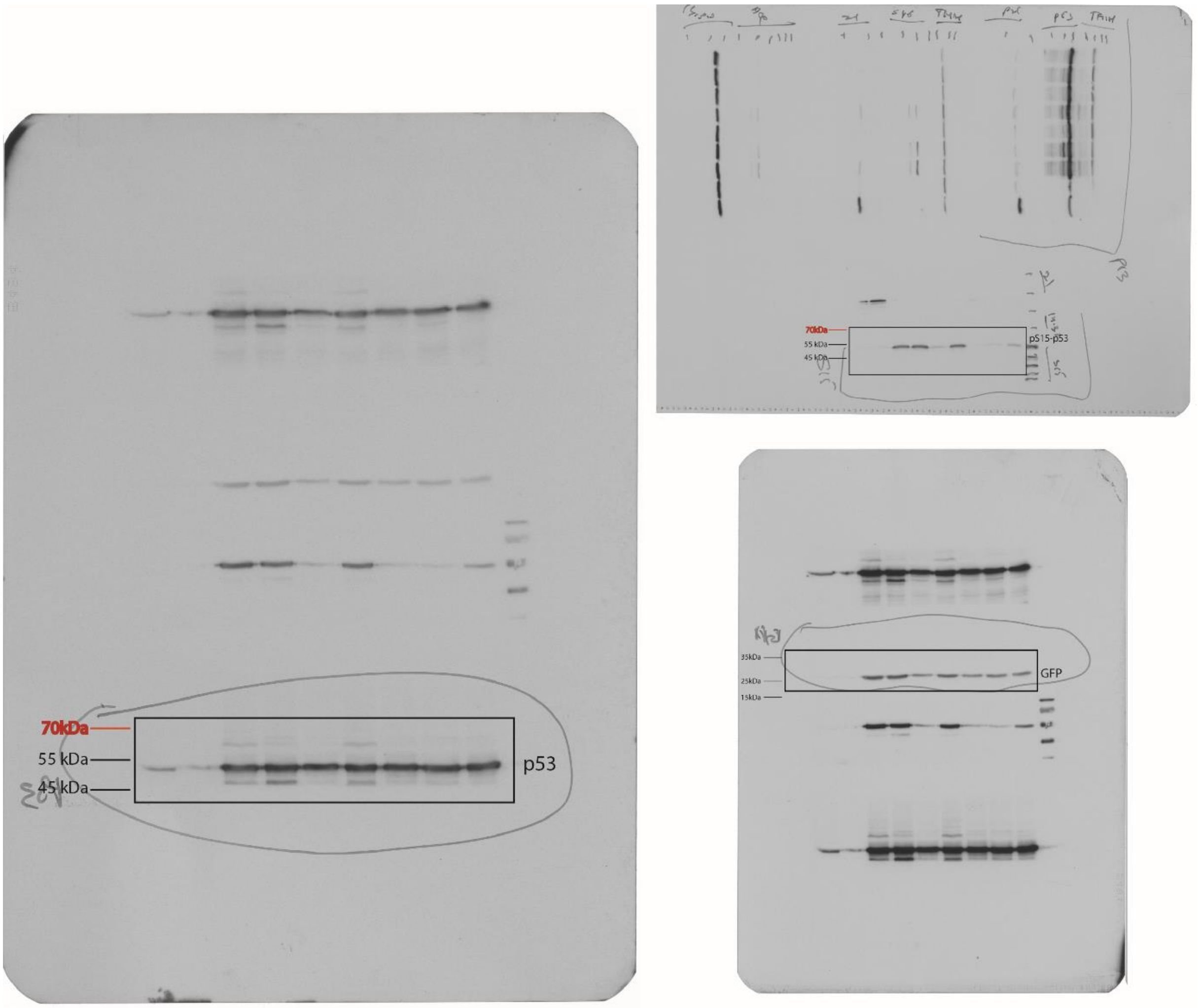

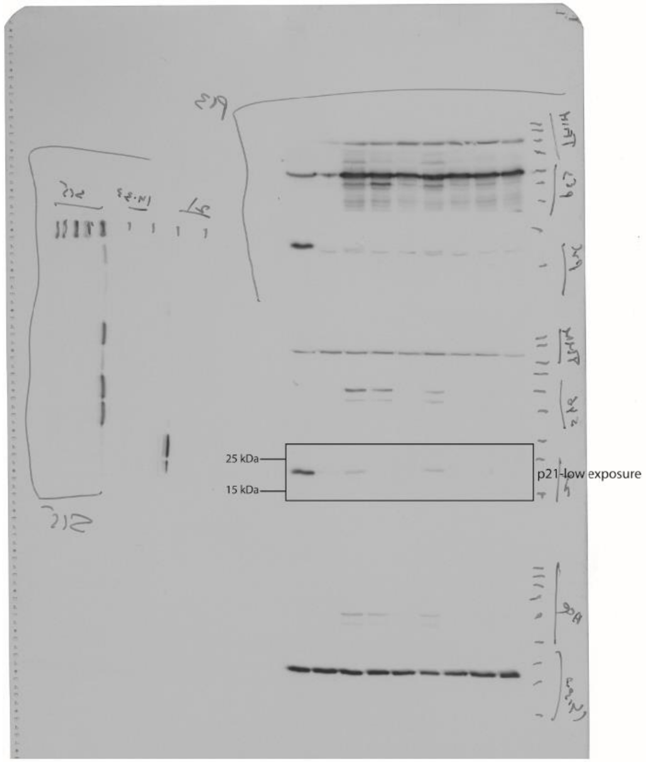
25 kDa
15 kDa
p21-low exposure

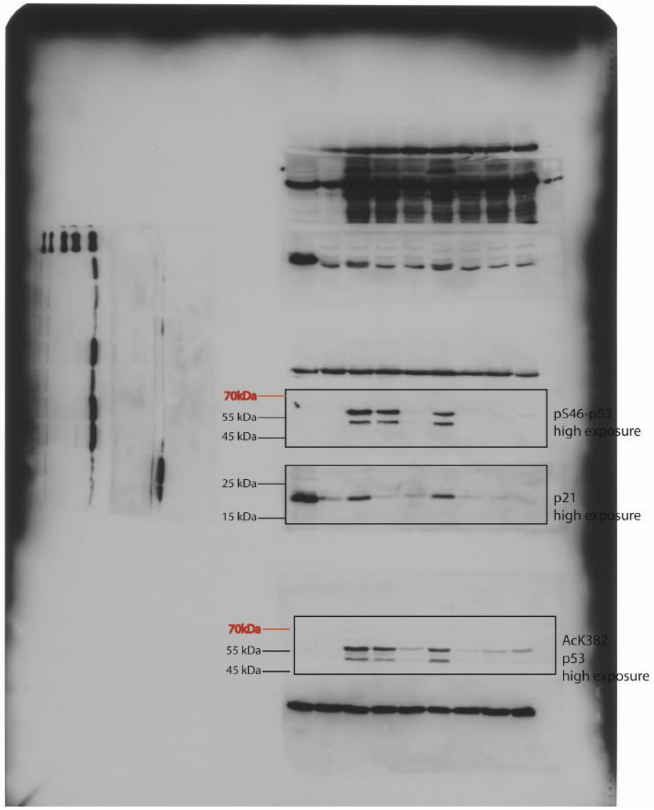
70kDa
55 kDa
45 kDa
pS46-p53
high exposure
25 kDa
15 kDa
p21
high exposure
70kDa
55 kDa
45 kDa
AcK382
p53
high exposure

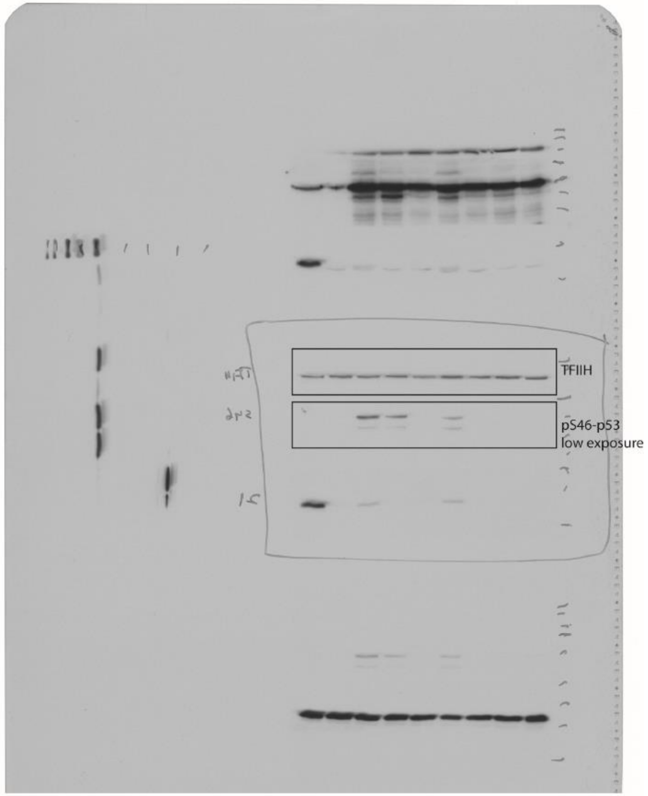
TFIIH
pS46-p53
low exposure
